# Preparation and Validation of a Longitudinally and Transversely Stiffened Panel Based on Hybrid RTM Composite Materials

**DOI:** 10.3390/ma16145156

**Published:** 2023-07-21

**Authors:** Weidong Li, Zhengzheng Ma, Pengfei Shen, Chuyang Luo, Xiangyu Zhong, Shicai Jiang, Weihua Bai, Luping Xie, Xiaolan Hu, Jianwen Bao

**Affiliations:** 1 National Key Laboratory of Advanced Composites, AVIC Composite Technology Center, AVIC Composite Corporation Ltd., Beijing 101300, China; liwdhappy@163.com (W.L.); 15364925153@163.com (Z.M.); 19821277882@163.com (P.S.); xyzhong2003@sohu.com (X.Z.); jiang_shicai@163.com (S.J.); 2 Collaborative Innovation Center for Civil Aviation Composites, Donghua University, Shanghai 201620, China; cyluo@dhu.edu.cn; 3 College of Materials, Xiamen University, Xiamen 361005, China; whuabai@163.com (W.B.); xieluping0806@163.com (L.X.)

**Keywords:** composite stiffened panel, resin-matrix composites, hybrid RTM, integral forming

## Abstract

In the face of the difficulty in achieving high-quality integrated molding of longitudinally and transversely stiffened panels for helicopters by resin-matrix composite materials, we combine the prepreg process and the resin transfer molding (RTM) process to propose a hybrid resin transfer molding (HRTM) for composite stiffened panel structures. The HRTM process uses a mixture of prepreg and dry fabric to lay up a hybrid fiber preform, and involves injecting liquid resin technology. Using this process, a longitudinally and transversely stiffened panel structure is prepared, and the failure modes under compressive load are explored. The results show that at the injection temperature of the RTM resin, the prepreg resin dissolves slightly and has little effect on the viscosity of the RTM resin. Both resins have good miscibility at the curing temperature, which allows for the overall curing of the resin. A removable box core mold for the HRTM molding is designed, which makes it convenient for the mold to be removed after molding and is suitable for the overall molding of the composite stiffened panel. Ultrasonic C-scan results show that the internal quality of the composite laminates prepared using the HRTM process is good. A compression test proves that the composite stiffened panel undergoes sequential buckling deformation in different areas under compressive load, followed by localized debonding and delamination of the skin, and finally failure due to the fracture of the longitudinal reinforcement ribs on both sides. The compressive performance of the test specimen is in good agreement with the finite element simulation results. The verification results show that the HRTM process can achieve high-quality integrated molding of the composite longitudinally and transversely stiffened panel structure.

## 1. Introduction

Advanced resin-matrix composite materials have been widely used in the aerospace field due to their high specific strength, high specific modulus, excellent fatigue performance, and design flexibility, which effectively reduce the weight of structures [1,2,3,4,5]. As a lightweight and high-strength structural component, composite stiffened panels have become the main load-bearing structures for aircraft fuselages, wings, and tail wings [6,7,8]. The helicopter fuselage typically adopts a longitudinally and transversely stiffened panel structure, combining tall and sparse ribs with low and dense ribs [9,10]. If traditional autoclave molding or resin transfer molding (RTM) is used for such composite stiffened panel structures, there will be issues such as high cost, low production efficiency, and uneven parts. The same qualified resin transfer molding (SQRTM) process [11,12], which evolved from traditional integral molding processes, has improved in terms of dimensional accuracy, overall molding performance, and production efficiency, making it an efficient molding process for fabricating composite stiffened panel structures. Even so, when using SQRTM to mold such longitudinally and transversely stiffened panel structures, difficulties may arise in the closing die of the web and the resin flow between the longitudinal and transverse ribs.

In addition, the failure modes of composite stiffened panels under load are complex. Under tensile and compressive loads, the composite stiffened panels experience debonding, delamination, and fiber fracture [13,14]. Moreover, under compressive loads, the composite stiffened panels also initially undergo buckling. Delamination of the reinforced rib plays a significant role in the strength of the stiffened panel, and subsequent fiber fracture leads to the ultimate failure of the composite stiffened panel. By studying the failure modes of the composite stiffened panels under tensile loads [15], compressive loads [16,17,18], shear loads [19,20,21], and with preexisting delamination defects [22,23,24], it is possible to use these failure modes to guide process improvements. Furthermore, investigating the effects of different process steps [13], process parameters [25,26], and fabric ply angles [27,28] on the performance of the composite stiffened panels can provide insights for improving process methods, enhancing molding quality, and optimizing structural design.

To address the challenges in forming such composite stiffened panels, we proposed a hybrid resin transfer molding (HRTM) process, specifically the prepreg–RTM co-molding process, to solve the molding issues of longitudinally and transversely stiffened panel structures for helicopters. The key to the HRTM process lies in using dry fabric for laying up the longitudinal and transverse ribs in the preform, while the skin is covered with prepreg material. This process involves mixing the prepreg with the dry fabric to form a hybrid preform, and then injecting liquid resin into the mold cavity to achieve co-curing. We have studied the compatibility between the prepreg resin and the RTM resin, designed and fabricated an integrated mold, produced a composite longitudinally and transversely stiffened panel, and explored its failure mode under compressive load, thereby validating the feasibility of the HRTM process for forming such stiffened panels. Combined with our preliminary validation results [29,30], this technology holds promise in addressing the challenges of traditional integral molding processes, thereby providing the possibility of achieving high-precision integrated molding for longitudinally and transversely stiffened panel structures by resin-matrix composites.

## 2. Results

### 2.1. Structural Design of the Composite Longitudinally and Transversely Stiffened Panel Based on HRTM

Researchers have been continuously developing advanced composite manufacturing technologies by combining integral molding and low-cost design techniques. The use of integral molding processes to produce composite stiffened panels is crucial for optimizing aircraft design and enhancing the competitiveness of composites in the aerospace field [31,32]. For example, the autoclave process has been used to pre-cure or fully cure the reinforcing ribs, followed by co-curing/co-bonding techniques to assemble the ribs and skin together for curing, achieving the integral molding of large-area composite components such as wing or fuselage skin [33,34]. This method involves co-curing or multiple curing steps, as well as secondary bonding, resulting in high costs, long process cycles, difficulties in controlling curing deformation, and poor dimensional accuracy. Liquid molding techniques also achieve high-precision integrated fabrication of complex composite structures [35,36,37]. However, for longitudinally and transversely stiffened panel structures, the resin flow field at the intersection of longitudinal and transverse ribs tends to generate turbulence, leading to defects such as dry spots or voids [38]. The SQRTM process combines the features of RTM and prepreg and is a closed-mold process developed in recent years for producing net-shape, highly integral composite parts. However, when forming longitudinally and transversely stiffened panel structures using SQRTM, the longitudinal and transverse ribs are covered with prepreg, requiring multiple prepressing steps to facilitate subsequent mold closing, resulting in complex molds and process difficulties.

**Figure 1:**
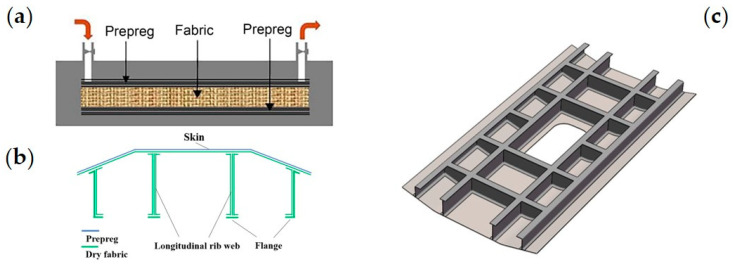
Composite longitudinally and transversely stiffened panel: (**a**) forming principle diagram of the HRTM process; (**b**) layup composition of the parts; and (**c**) model drawing of the composite stiffened panel structure.

The HRTM process that we propose combines the advantages of prepreg and RTM techniques. In this process, we design the longitudinal and transverse ribs to be laid with dry fabric in the preform, while the skin is covered with prepreg. The prepreg of the skin is used to improve the bearing capacity of the structure. The use of dry fabric for longitudinal and transverse reinforcement is because the RTM preform has compression deformation under the mold-closing force, which can solve the problem of mold closing and improve the internal quality of the composite structure. This involves the mixture of prepreg and dry fabric in the preform, followed by injecting the RTM resin into the mold cavity. This allows for the co-curing of prepreg and RTM during the molding process. The forming principle diagram of the HRTM process is shown in Figure 1a.

An epoxy/carbon fibric (AC531/CCF800H) prepreg (from AVIC Composite Corporation Ltd., Beijing, China, with a surface density of 145 g/m^2^, resin content of 35%, and ply thickness of 0.140 mm) and a U-8190 unidirectional dry carbon fabric (from AVIC Composite Corporation Ltd., with a surface density of 190 g/m^2^, tackifier content of 6%, and ply thickness of 0.200 mm) are used to fabricate the fiber preform. The HRTM process is then carried out using low-viscosity 5284 RTM epoxy resin (from AVIC Composite Corporation Ltd.). The layup composition of the component is shown in Figure 1b. The skin is made up of eight layers of prepreg and seven layers of fabric. The reinforcement is made up of 15 layers of fabric. The middle opening part consists of 8 layers of prepreg and 12 layers of fabric. The opening part was strengthened locally. The model of the helicopter fuselage component is depicted in Figure 1c.

### 2.2. Feasibility of the HRTM Process

**Figure 2:**
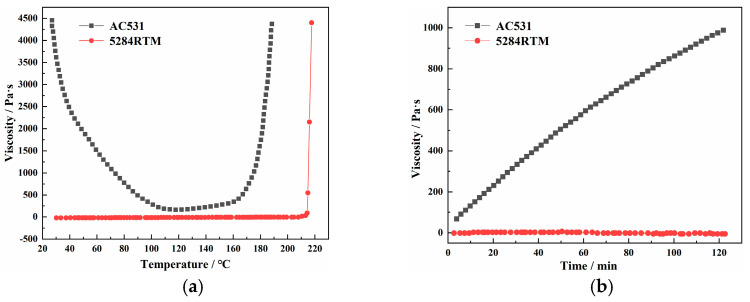
Rheological curves of AC531 prepreg epoxy resin and 5284 RTM epoxy resin: (**a**) rising temperature; and (**b**) constant temperature at 100 °C.

The compatibility between the prepreg resin and the RTM resin is crucial for the implementation of the HRTM process. We selected low-viscosity 5284 RTM epoxy resin and high-viscosity AC531 prepreg epoxy resin with high toughness as the resin matrix. Initially, we investigated the compatibility between the two resins to assess the feasibility of HRTM. Using an AR2000 rheometer (Thermal Analysis & Rheology Instruments Inc., New Castle, DE, USA), we performed temperature ramp rheology tests (ramp rate of 2 °C/min) and isothermal rheology tests (at 100 °C) for both resins. The results are shown in Figure 2. We found that at room temperature, the viscosity of the AC531 resin was significantly higher than that of the 5284 RTM resin. As the temperature increased, the viscosity of the AC531 resin rapidly decreased. At 110 °C, the AC531 resin entered a low-viscosity region. However, the viscosity of the AC531 resin remained higher than that of the 5284 resin. Based on the preliminary results in Figure 2a, a temperature range of 100–110 °C was identified as the HRTM process window. The viscosity curves of the two resins at 100 °C are shown in Figure 2b. It can be observed that with increasing isothermal time, the viscosity of the AC531 resin increased rapidly, while the viscosity of the 5284 resin remained almost constant within the range shown in the graph. This suggests that during the co-curing process, the flowability of AC531 prepreg resin gradually deteriorated, and it can be considered to have limited flowability.

**Figure 3:**
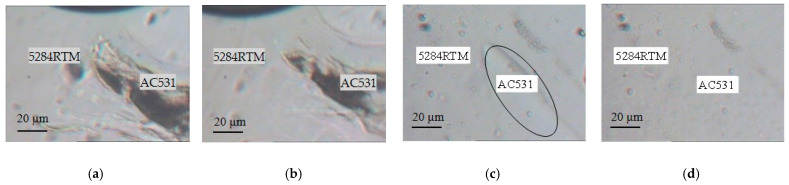
The miscible process of 5284 RTM epoxy resin and AC531 prepreg epoxy resin: (**a**) 100 °C /0.5 h; (**b**) 100 °C /9.5 h; (**c**) 180 °C /0 h; and (**d**) 180 °C /0.5 h.

Utilizing a self-designed inverted optical microscope with a heating stage, we observed the interfacial diffusion process between the two resins at an injection temperature of 100 °C. We found that after 0.5 h at 100 °C, there was no significant diffusion of the AC531 prepreg resin into the 5284 RTM resin (Figure 3a). This indicates that at low temperature for short duration, there was no significant interdiffusion or dissolution between the two resin phases. However, after 9.5 h at 100 °C, the AC531 prepreg resin was slightly dissolved by the 5284 RTM resin (Figure 3b). The injection process in RTM process typically takes place within 5 h, which means that the entire co-curing process would be completed before significant dissolution of the prepreg resin occurs. Therefore, the partial dissolution of the prepreg resin would not affect the RTM process.

We further explored the compatibility between the two resins during the curing process. As the temperature was raised to a co-curing temperature of 180 °C, partial mutual dissolution between the two resin phases occurred, as shown in Figure 3c. The interface between the two phases was clearly observed. After 0.5 h at 180 °C (Figure 3d), the two resins completely merged, and the interface disappeared. This indicates that the two resins can be compatible during the curing stage, leading to the formation of a cohesive material.

### 2.3. Finite Element Analysis of the Composite Stiffened Panel Based on HRTM

**Figure 4:**
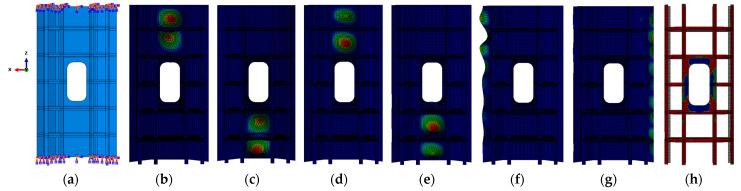
Finite element analysis of composite stiffened panel: (**a**) load and boundary condition setting of finite element model; (**b**–**g**) the first six buckling modes of the stiffened panel structure; and (**h**) the postbuckling mode of the stiffened panel structure.

The commercial finite element analysis software ABAQUS (2016) was employed to model the effective section of the experimental model and understand the performance of the composite stiffened panel. Due to the significantly smaller thickness of the stiffened panel compared to other dimensions (less than 1/10), and the negligible stress in the thickness direction, the skin and stiffeners of the stiffened panel were simulated using conventional shell elements, specifically the S4R element. The applied loads and boundary conditions are shown in Figure 4a, where a fixed support boundary condition was applied at the bottom to simulate the constraint at the glued region, and a downward compressive load was applied at the top while constraining the degrees of freedom in other directions. The lower end support constraint, that is, the six degrees of freedom (x, y, z translational and rotational degrees of freedom around the three axes) were constrained. The upper end was constrained by 5 degrees of freedom, and the freedom of axial loading was released, that is, y, z translation and 3 rotational degrees of freedom. Note that the x direction is the loading direction, and the upper end is not constrained in the x direction, allowing the structure to have displacement in the x direction. The material parameters for the skin and stiffeners used in the finite element simulation are listed in Table 1.

We used the 2D Hashin failure criteria to assess material failure. This criteria distinguishes four types of damage modes: fiber tension, fiber compression, matrix tension, and matrix compression. Abaqus has built-in support for these failure criteria, with a default value of 0.0001 for the interlaminar shear strength. The failure criteria equations are as follows:

Failure criterion of fiber tension:

(1) $${\left(\frac{\sigma_{1}}{X_{T}}\right)}^{2}+{\left(\frac{\tau_{12}}{S_{12}}\right)}^{2}=1\;\mathrm{for}\;\sigma_{1}>0$$

Failure criterion of fiber compression:

(2) $$\left|\sigma_{1}\right|=X_{C}\;\mathrm{for}\;\sigma_{1}<0$$

Failure criterion of matrix tension:

(3) $${\left(\frac{\sigma_{2}}{Y_{T}}\right)}^{2}+{\left(\frac{\tau_{12}}{S_{12}}\right)}^{2}=1\;\mathrm{for}\;\sigma_{2}>0$$

Failure criterion of matrix compression:

(4) $${\left(\frac{\sigma_{2}}{2S_{12}}\right)}^{2}+\left[{\left(\frac{Y_{C}}{2S_{12}}\right)}^{2}-1\right]\frac{\sigma_{2}}{Y_{C}}+{\left(\frac{\tau_{12}}{S_{12}}\right)}^{2}=1\;\mathrm{for}\;\sigma_{2}<0$$

where *σ*_1_: one-directional stress; *σ*_2_: two-directional stress; and *τ*_12_: in-plane shear stress.

Buckling is a stiffness problem. According to linear buckling theory, a structure always reaches equilibrium in its unloaded initial configuration. When buckling occurs, the structure’s configuration abruptly transitions from one equilibrium state to another. The response of the structure after buckling is referred to as postbuckling. After reaching the buckling load, the structure does not immediately undergo failure but still possesses a certain load-carrying capacity in the postbuckling regime. The buckling load of the structure can be obtained according to the following formula:

(5) $$F_{Buckle}=K\times U_{Buckle}$$

where $F_{Buckle}$ is the buckling load, K is the stiffness of the laminate, and $U_{Buckle}$ is the buckling displacement. The buckling displacement can be calculated as follows:

(6) $$U_{Buckle}=\lambda \times U_{load}$$

where λ is the characteristic value obtained from buckling analysis. Since the load of the first order buckling mode was to be obtained, the characteristic value of the first order mode is used for calculation. $U_{load}$ is the axial displacement loaded to the model. Equations (1)–(4) can be used to predict the failure load of postbuckling, and Equations (5) and (6) can be used for buckling analysis to obtain buckling load and buckling mode. The failure mode and failure strength of the stiffened panels are obtained by finite element analysis to ensure that the designed structure meets the design requirements.

In Abaqus, the front-buckling analysis is performed using the linear buckling module “Buckle”. Firstly, a unit axial displacement is applied to obtain the first eigenvalue, which represents the buckling displacement. Then, a static load analysis is conducted by applying the buckling displacement to determine the support reactions at the loaded edges, which correspond to the buckling load. The buckling analysis reveals the first six buckling modes of the model, as shown in Figure 4b–g. It can be observed that local buckling occurs in the skin of the stiffened panel. The critical buckling load is determined to be 158 kN. Subsequently, a postbuckling analysis is performed to consider the nonlinear effects. A 1% thickness as an initial defect is introduced into the model to simulate the entire failure process. The Hashin failure criteria and progressive failure evolution are defined for the composite laminate to simulate the debonding at the interface between the skin and the stiffeners (Figure 4h). The structure undergoes failure when the load reaches 365 kN.

### 2.4. Mold Design of the Composite Stiffened Panel Based on HRTM

**Figure 5:**
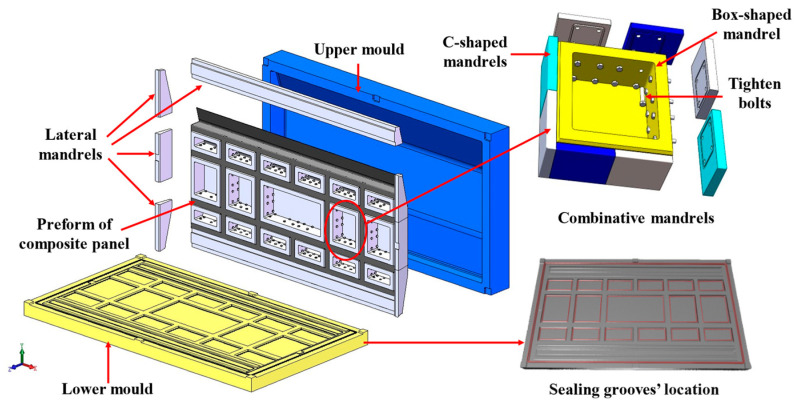
Mold diagram of the composite stiffened panel [39].

The mold is a key factor in achieving high-quality molding of the stiffened panel structure using the HRTM process. The HRTM mold requires good sealing performance and needs to be designed with appropriate mold-opening and -closing mechanisms, demolding mechanisms, sealing systems, flow systems (including inlet and outlet ports), and corresponding auxiliary tooling that aligns with the component structure. To achieve the integrated molding of the stiffened panel structure and avoid the problem of the mold becoming stuck after forming the “T-shaped” reinforced structure, we designed a combination core mold, as shown in Figure 5. The uncovered portion of the “T-shaped” structure is designed as a box-type core mold that can be freely removed. During the demolding process, the box-type core mold is first removed, followed by sequentially removing the C-shaped forming mold in a direction perpendicular to the belly panel.

### 2.5. Internal Quality of the Composite Laminates Based on HRTM

**Figure 6:**
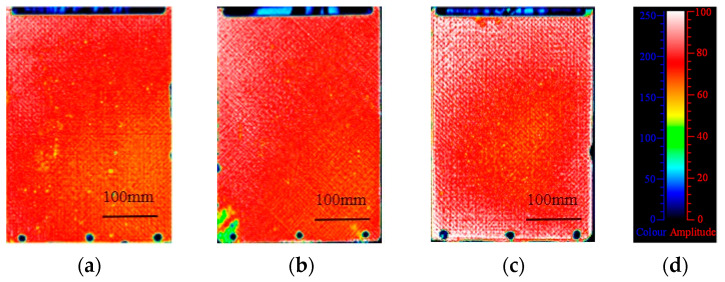
Composite laminates by the HRTM process: (**a**–**c**) ultrasonic C-scan images; and (**d**) colorimetric card.

We used the HRTM process to manufacture composite laminates and preliminarily verified the feasibility of the HRTM process by evaluating the internal quality of the laminates. Ultrasonic C-scan is a commonly used nondestructive testing technique for composite materials, which can detect internal defects. The results of the ultrasonic C-scan and a color chart for comparison are shown in Figure 6. In order to check repeatability, we prepared five composite laminates using the HRTM process. The bottom echoes of each laminate are high, with amplitudes greater than 80%, indicating low material attenuation and good internal quality of the manufactured laminates.

### 2.6. Composite Longitudinally and Transversely Stiffened Panel Based on HRTM

**Figure 7:**
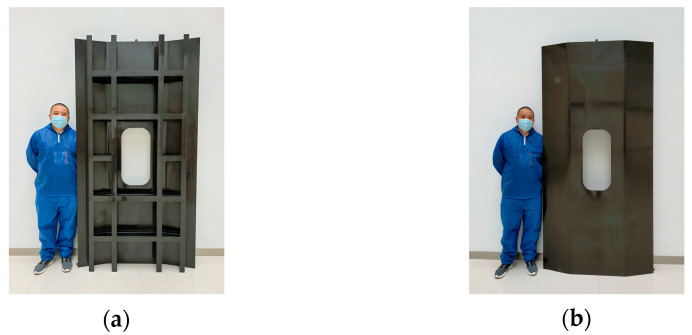
Composite longitudinally and transversely stiffened panel by HRTM: (**a**) stiffening surface; and (**b**) skin surface.

The HRTM process includes preforming, layup, molding, resin injection, curing, and demolding. Firstly, the C-shaped core and box-shaped core are assembled into a combined core mold as shown in Figure 5. Dry fabric is laid up on the combined core mold, and vacuum preforming is conducted at 60 °C for 1 h. Unidirectional prepreg is used for skin layup. The temperature of the mold and resin injection tank is raised to 100 °C, and 5284 RTM epoxy resin is injected into the mold until the resin completely impregnates the structure. The mold temperature is then raised to 180 °C at a rate of 5 °C/min and maintained for 2 h for curing. After natural cooling of the mold to room temperature, demolding is performed, followed by machining and edge trimming, resulting in a composite stiffened panel with longitudinal and transverse reinforcements as shown in Figure 7.

**Figure 8:**
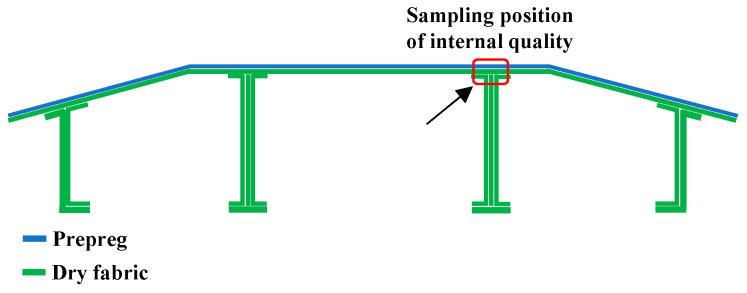
Typical part of internal molding quality selection.

**Figure 9:**
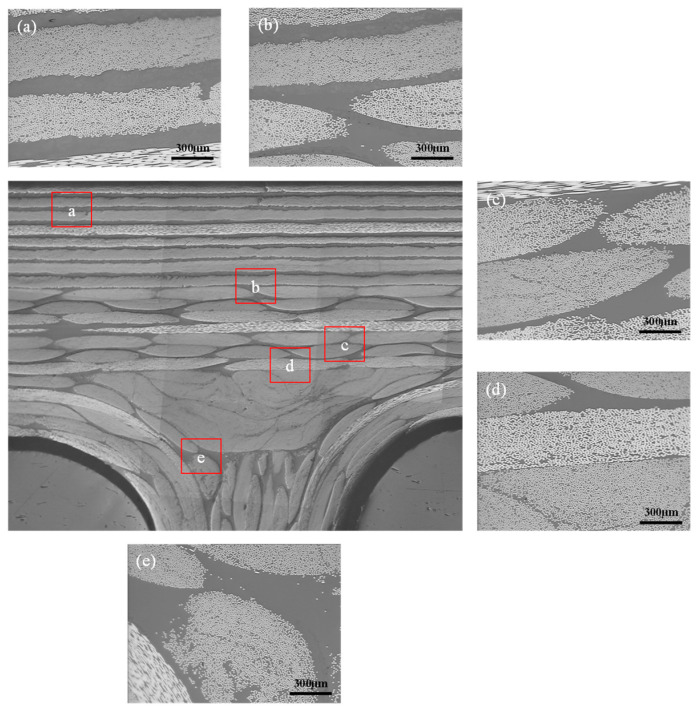
Optical microscope images of the typical area. (**a**) the prepreg composite material region; (**b**) the overlap region between prepreg and fabric; (**c**) the fabric composite material region; (**d**) the overlap region between fabric and triangular fillers; (**e**) the longitudinal reinforcement composed of the triangular fillers and fabric.

The application of RTM in the manufacturing of longitudinally and transversely stiffened panel structures often leads to turbulent flow at the intersection of the longitudinal and transverse reinforcements, which can result in defects such as dry spots or dense voids [29]. In order to observe the microstructure of the corresponding areas in the composite stiffened panel, we selected the most challenging location, which is the integration area between the panel skin and the longitudinal reinforcement (Figure 8), to investigate the internal quality of the composite stiffened panel. The optical microscope images of the typical area are shown in Figure 9. We examined five different positions. Position (a) represents the prepreg composite material region. Position (b) represents the overlap region between prepreg and fabric. Position (c) represents the fabric composite material region. Position (d) represents the overlap region between fabric and triangular fillers. Position (e) represents the longitudinal reinforcement composed of the triangular fillers and fabric. From Figure 9, it can be observed that the internal quality in the selected typical area is good, and no void defects are detected.

### 2.7. Performance Verification of the Composite Stiffened Panel

**Figure 10:**
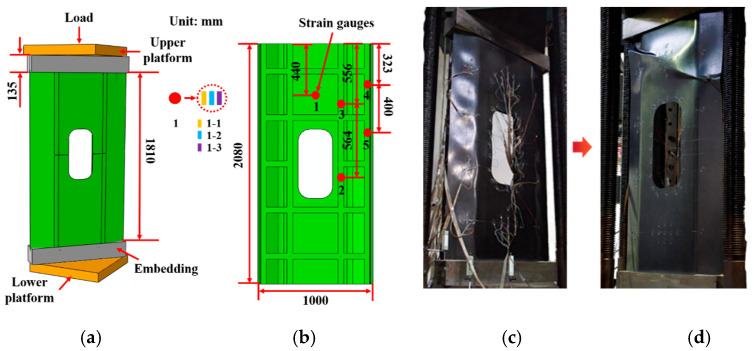
Compression test of the composite stiffened panel: (**a**) loading method; (**b**) position of strain gauge; (**c**) local buckling of skin; and (**d**) final failure.

We conducted compressive tests on the stiffened panel to assess its mechanical performance. The stiffened panel was machined into a compression test specimen with dimensions of 2080 mm × 1000 mm. The ends of the specimen were sealed with resin for a length of 135 mm, as shown in Figure 10a. The JM3813 static strain measurement system (Yangzhou Jingming Technology Co., Ltd., Yangzhou, China) was used to measure the strain of the stiffened panel during loading. BE120-3AA resistance strain gauges (AECC Measurement & Control Technology Co., Ltd., Beijing, China) were used as the strain sensors. Unidirectional strain gauges were attached along the loading direction, and the arrangement of the strain gauges is shown in Figure 10b. Meanwhile, the dimensions of the structure are given in Figure 10b. The skin thickness is 2.54 mm, of which 1.12 mm is AC531/CCF800H prepreg composite material and 1.40 mm is 5284RTM/U-8190 fabric composite material. The reinforcement is 3.0 mm thick and is made of 5284RTM/U-8190 fabric composite. Due to the relatively large area of the strain measurement locations, three strain gauges were attached to each strain measurement location to ensure accurate and reliable experimental data.

**Figure 11:**
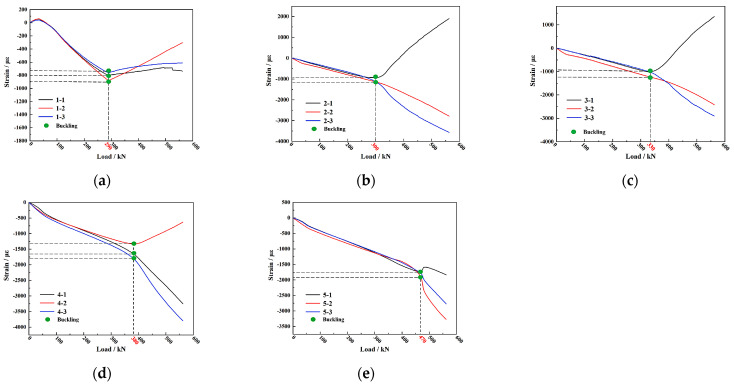
Load–strain curves of the stiffened panel in compression test: (**a**) skin in pellet area (no. 1 strain); (**b**) reinforcement bars in open area (no. 2 strain); (**c**) middle reinforcement bars in nonopen area (no. 3 strain); (**d**) double reinforcement bars (no. 4 strain); and (**e**) double reinforcement bars (no. 5 strain).

We used a 500 ton hydraulic testing machine (Jinan Gold Test Group Co., Ltd., Jinan, China) to apply the load at a rate of 0.1 kN/s, with strain data collected every 2 kN. When the load reached 65 kN, the stiffened panel produced slight noise but showed no significant deformation. As the load increased to 290 kN, the load–strain curve of the core skin changed from linear to nonlinear and even exhibited a reversal (Figure 11a), indicating the occurrence of local buckling in the skin of each core section [40]. Figure 10c clearly shows the initial occurrence of local buckling in the skin during loading. When the load further increased to 300 kN, the skin near the opening area and the longitudinal rib web experienced successive local buckling (Figure 11b). As the load increased, the stiffened panel continuously emitted noise. At 330 kN, the non-opening area of the two central longitudinal rib webs started to buckle and deform (Figure 11c). When the load reached 380 kN, the side ribs began to buckle and deform (Figure 11d), leading to the maximum buckling load of 470 kN (Figure 11e). 

In the test, the load–strain curve of the buckling part and the test video was used to identify whether buckling occurred. Due to the fact that the cutout is prone to buckling in the structure, local reinforcement was carried out in the cutout site. The main reason for this failure sequence is the lack of reinforcement protection in the skin areas, resulting in the initial occurrence of buckling deformation. Additionally, due to the lack of transverse reinforcement support in the skin and reinforcement at the opening area, poor structural continuity led to buckling deformation after the core skin buckled. The nonopening area of the ribs, being away from the opening area, had a complete structure and simultaneous compression load on the longitudinal and transverse reinforcements, resulting in buckling deformation occurring last. When the load reached 565 kN, a loud noise was heard from the stiffened panel. During continuous loading, similar noises were produced at loads of 568 kN and 571 kN, which may be attributed to delamination of the skin or debonding between the skin and the reinforcement after buckling deformation of the stiffened panel. When the load increased to 575 kN, a loud sound was emitted, and the structure experienced more than 30% loss of load-bearing capacity, indicating structural failure. The overall appearance of the specimen after failure is shown in Figure 10d.

**Figure 12:**
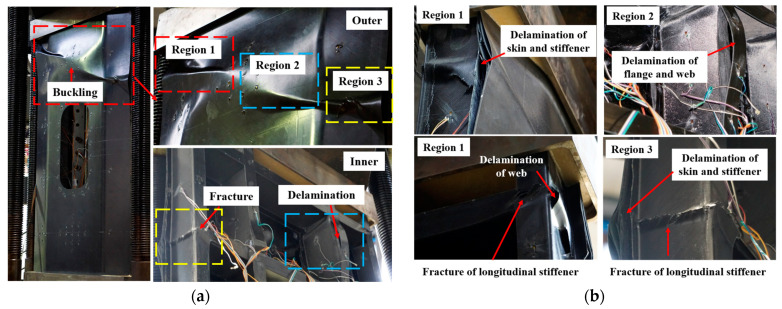
Failure regions of the composite stiffened panel after compression test: (**a**) overall failure morphologies; and (**b**) detail failure morphologies at regions 1–3.

Observing the morphology of the stiffened panel after the compression test (Figure 12), we found that the major damages were concentrated in the upper half of the specimen, while no significant damage was observed in the lower half of the specimen. The outer surface of the skin exhibited primarily wrinkling caused by buckling failure, while the internal surface of the stiffened panel experienced two types of damage, namely, delamination and fracture of the reinforcements. Based on the extent of damage, we divided the failure locations into three regions, as shown in Figure 12a. Region 1 (Figure 12b) exhibited delamination and debonding between the skin and the reinforcements, as well as significant fracture of the internal reinforcements. The maximum strain in the reinforcements at this location is −3797 με (Figure 11d), whereas the maximum strain in the core skin throughout the entire experiment is only −884 με (Figure 11a). This indicates that the wrinkles in region 1 were a result of the energy and displacement generated by the fracture of the reinforcements after buckling, leading to delamination and debonding of the skin. In region 2, the internal reinforcements showed debonding and fractures at the intersection of the longitudinal and transverse reinforcements. The maximum strain on the reinforcements in this region is −2900 με (Figure 11c). Due to the presence of transverse reinforcements, the fractures were not as prominent. The failure morphology in region 3 was similar to region 1, with fractures occurring in the reinforcements and noticeable delamination and separation of the skin.

Under the compression load, although the composite stiffened panel exhibited buckling deformation at various locations as the load increased and the buckling deformation of the core skin was prominent, the ultimate failure primarily occurred in the upper half of the stiffened panel. The failure modes mainly included skin debonding, delamination, and reinforcement fracture. It is evident that the stiffened panel maintained a high load-bearing capacity even under skin buckling until the longitudinal reinforcements fractured, resulting in overall structural failure. It is important to note that in this test specimen, the debonding between the skin and the reinforcements was mainly caused by the sudden release of energy during reinforcement fracture, which differs from the rib debonding failure observed in traditional reinforced stiffened panel structures.

The failure of the test specimen occurred due to the crushing of the reinforcement web, rendering the structure unable to bear the load. The extensive delamination between the skin and the reinforcements was caused by the instantaneous release of energy during fracture, consistent with the results obtained from finite element simulations. The failure load of the specimen was determined to be 575 kN. The buckling load of the specimen was obtained from the load–strain curve. At 280 KN, all grid skins exhibited buckling deformation. Continuing to load up to 300 KN, the two central longitudinal reinforcement webs began to deform. At 330 kN, 380 kN, and 450 kN, the different reinforcement webs successively underwent buckling deformation. The results from finite element simulations significantly differed from the actual failure load. Taking into account the continuous sound and the buckling deformation of both the skin and the webs at 380 kN, which indicated structural debonding, a comparison was made between 380 kN and the finite element results, resulting in a difference of 6%.

The composite stiffened panel structure does not suffer buckling failure under the axial compression load of 150 kN (150% of the service load). Based on the above analysis, it can be concluded that the proposed HRTM process achieved high-quality integrated forming of the longitudinal and transverse stiffened panel with resin-matrix composite materials. The integration between the longitudinal and transverse reinforcements requires no additional connecting structures, ensuring good load transfer between the reinforcements and the skin. The connection strength between the reinforcements and the skin is excellent, providing the structure with outstanding load-bearing capacity.

## 3. Conclusions

Combining the characteristics of the prepreg process and the resin transfer molding (RTM) process of resin-matrix composite materials, we proposed a hybrid RTM (HRTM) process for the integrated forming of composite stiffened panel structures for helicopters. The HRTM process involves the combination of prepreg and dry fabric layers, with dry fabric used for the longitudinal and transverse reinforcement ribs and prepreg used for the skin. In the HRTM process, the prepreg epoxy resin and the RTM epoxy resin met the conditions during the resin injection stage and exhibited good miscibility during the curing stage, resulting in a cohesive resin matrix after curing. We designed a demoldable box-type core mold structure based on the HRTM process, facilitating easy mold removal after forming and suitable for the integrated forming of the composite stiffened panel. Composite laminates were fabricated using the HRTM process, and nondestructive testing results indicated excellent internal quality. Subsequently, a composite stiffened panel structure was fabricated using the HRTM process, and under compressive loads, the structure exhibited sequential buckling deformations, followed by localized debonding and delamination of the skin, and ultimately failure due to the fracture of the two longitudinal reinforcement ribs. The composite stiffened panel structure fabricated using the HRTM process exhibited good internal quality without defects such as dry spots or dense voids at the intersections of the reinforcement webs. The compression performance of the test specimens is in good agreement with finite element simulation results. The HRTM process enables the achievement of good load transfer between the reinforcement webs and the skin without the need for additional connecting structures, ensuring the high-quality integrated forming of reinforced stiffened panel structures.

## Figures and Tables

**Table 1:** Material property parameters.

Items	Average Value	Standard Deviation	Dispersion Coefficient, %	Number of Samples
*E*_1T_/GPa	164.3	4.54	2.92	5
*E*_2T_/GPa	8.52	0.086	1.12	5
*G*_12_/GPa	4.45	0.12	2.71	5
*υ*_12_	0.32	0.009	2.65	5
*X*_T_/MPa	2536	103	4.18	5
*X*_C_/MPa	1523	75.3	4.51	5
*Y*_T_/MPa	66.50	3.40	4.57	5
*Y*_C_/MPa	194.3	15.2	4.26	5
*S*_12_/MPa	110	2.71	1.74	5

Notes: *E*_1T_—longitudinal elastic modulus; *E*_2T_—transverse elastic modulus; *G*_12_—1,2 direction shear modulus; *υ*_12_—1,2 direction Poisson’s ratio; *X*_T_—longitudinal tensile strength; *X*_C_—longitudinal compressive strength; *Y*_T_—transverse tensile strength; *Y*_C_—transverse compressive strength; *S*_12_—in-plane shear strength.

## Data Availability

Not applicable.
